# Toxic Adenoma in a Patient with Thyroid Hemiagenesis

**DOI:** 10.7759/cureus.1695

**Published:** 2017-09-17

**Authors:** Emin Gurleyik, Sami Dogan, Fuat Cetin, Fatih Gursoy, Alper M Ipor

**Affiliations:** 1 Department of Surgery, Duzce University Medical Faculty

**Keywords:** congenital anomaly, agenesis, hyperthyroidism, surgery

## Abstract

Thyroid hemiagenesis (TH) is a rare congenital anomaly that is usually asymptomatic. Functional disorders of the thyroid make the patient symptomatic. TH is usually and incidentally established during evaluation of patients with symptomatic thyroid pathology. We report the case of a patient of TH who became symptomatic with hyperactivity of the gland. The patient presented with signs and symptoms of thyrotoxicosis. Physical examination revealed asymmetric nodular goiter at the right lobe. Biochemical analysis established the diagnosis of hyperthyroidism. Ultrasound of the thyroid gland revealed the absence of the left lobe and a large, solitary hypoechoic solid nodule in the right lobe. Nuclear scan showed the absence of the left lobe and revealed a large, autonomous solitary nodule in the right lobe. The diagnosis was a toxic adenoma. After medical control of hyperthyroidism, the patient was surgically treated with hemithyroidectomy. We prescribed postoperative replacement medication with L-thyroxin. Hyperthyroidism makes TH cases symptomatic. Thyroid ultrasound and scintigraphy incidentally discover agenesis of one lobe during evaluation of thyrotoxicosis. Hemithyroidectomy, including the autonomous nodule, is the procedure of choice for patients with toxic adenoma. Hemithyroidectomy in TH cases technically becomes a total thyroidectomy with a need for postoperative thyroid replacement therapy.

## Introduction

Total absence of one lobe of the thyroid gland, thyroid hemiagenesis (TH), is a rare anatomical anomaly that is usually asymptomatic. The remaining lobe generally has a normal function, due to which TH is diagnosed accidentally by cervical imaging. The pathogenesis and clinical significance of this malformation remain undefined, and specific clinical recommendations are lacking, especially for asymptomatic cases. Therefore, discovering TH in asymptomatic cases is possible only by a screening program using various imaging modalities [1-4]. TH is usually established during evaluation of patients with symptomatic thyroid pathology. Functional disorders of the thyroid gland such as hyperactivity make the patient symptomatic [5-6]. This report presents TH discovered during evaluation of a patient with signs and symptoms of hyperthyroidism.

## Case presentation

**Figure 1:**
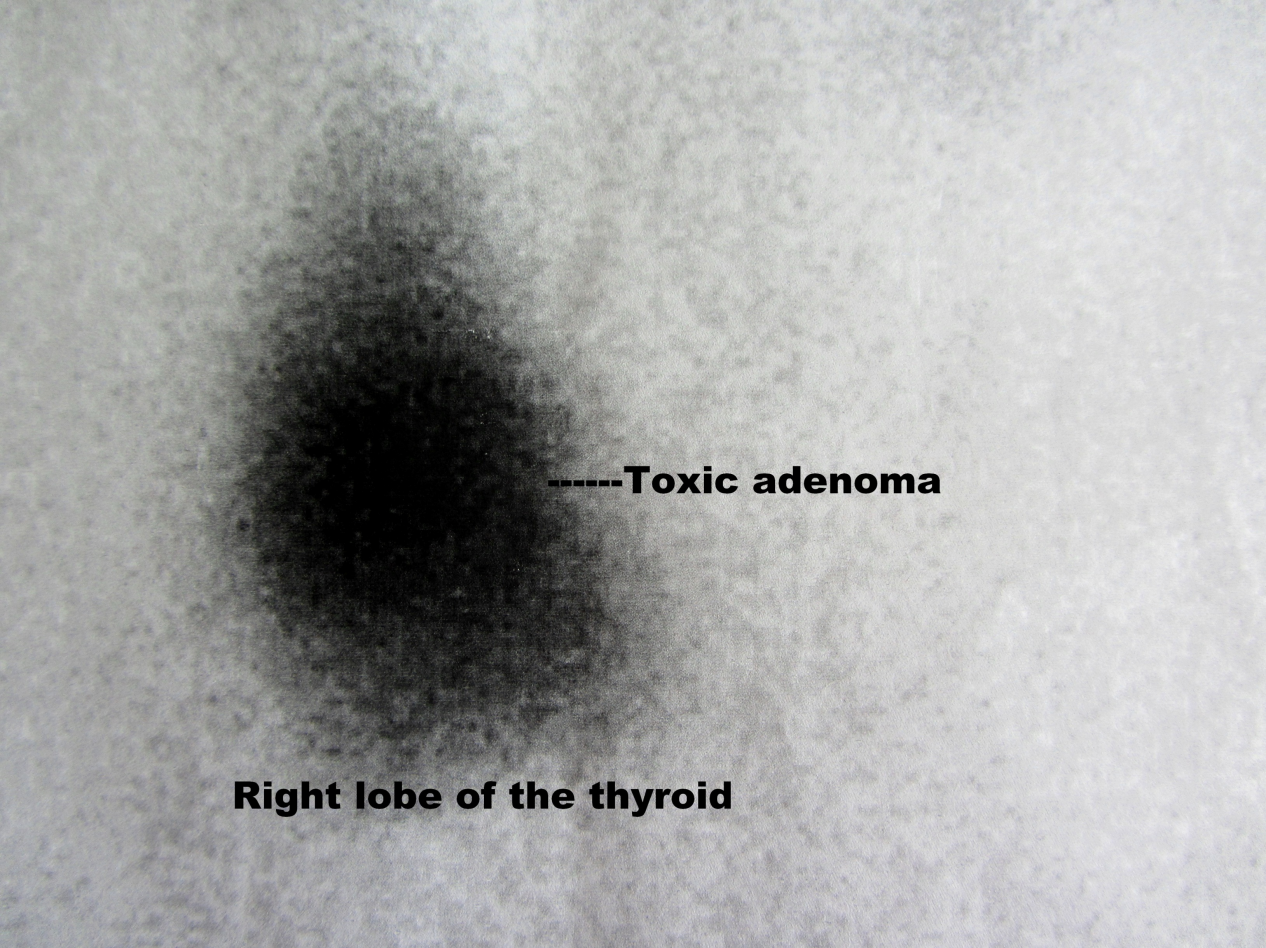
Nuclear scan of thyroid gland. It reveals hot, hyperactive, large solitary nodule in the right lobe. The left lobe is not visualized.

A 51-year-old woman presented to our clinic with signs and symptoms of hyperthyroidism. Asymmetric hypertrophy of the thyroid gland at the right side was determined by inspection. A larger nodule with regular margins was palpated in the right lobe by physical examination. Suppressed serum thyroid-stimulating hormone (TSH = 0.03 µIU/mL) and high free thyroxin (FT4 = 2.42 ng/dL) levels were determined by biochemical analysis. Ultrasound of the thyroid gland revealed the absence of the left lobe and a 33 × 25 mm hypoechoic solid nodule with a cystic component in the right lobe. Thyroid nuclear scan with Tc-99 m pertechnetate showed that the left lobe was not visualized (agenesis) and further revealed a larger, hot, autonomous, hyperactive solitary nodule in the right lobe (Figure 1). Cervical magnetic resonance imaging (MRI) revealed the absence of the left lobe and isthmus of the thyroid gland, and a 26 x 23 mm solid nodule with a central cystic component. The solitary nodule was iso-hypointense in T1AG and heterogeneously hyperintense in T2AG. The diagnosis was toxic adenoma in the right lobe in this patient with TH (absence of the left lobe). The patient preoperatively received antithyroid medical treatment with thyromazol. Antithyroid drugs were used until the operation under the control of thyroid function tests. We totally excised the remaining right lobe, including the large, hyperactive solid nodule. Right superior and inferior parathyroid glands and also the recurrent laryngeal nerve (RLN) were identified at the usual anatomical position. They were fully exposed and preserved during thyroid surgery. Pre- and postoperative laryngoscopy showed normal vocal cords functions. Thyroid surgery was performed under the guidance of intraoperative nerve monitoring that wave amplitudes after pre- and post-dissection stimulation of the vagus nerve (V1: 654 µV and V2: 1249 µV) and RLN (R1: 1652 µV and R2: 1750 µV) showed functional integrity of laryngeal nerves. Postoperative period was uneventful. The patient was discharged on the second postoperative day. The pathological report showed that the size of the right lobe is 50 × 40 × 35 mm, which contained a solid nodule (30 × 25 × 25 mm). The histopathological diagnosis was follicular nodular disease. The patient is euthyroid with LT4 (150 µg/day) replacement.

## Discussion

Human thyroid development is a complex and still unexplained process. TH is a rare congenital anomaly, where one of the thyroid lobes fails to develop [7]. The thyroid rudiment developed from the endoderm, grows laterally to create lateral lobes of the gland. Hemiagenesis is an incomplete genesis of a lobe for which the aetiology remains unclear [1,7]. Suzuki, et al. [3] examined approximately 300,000 children and young adults to determine the presence of thyroid agenesis or hemiagenesis. The thyroid width, thickness, and length were measured. Thyroid agenesis was diagnosed in 13 subjects, and hemiagenesis was detected in 67 (0.02%; 22.3 per 100,000 individuals) [3]. The prevalence of TH has been reported between 0.025% and 0.05% in normal population, and between 0.16% and 0.25% in patients with thyroid disorders [2]. It is interesting to note that most of the cases have an agenesis of the left lobe (80% of cases), followed by that of the isthmus (44%–50% of cases) [6]. Our patient is an example of left lobe absence associated with symptomatic functional disorders of the remaining right lobe. The case is a middle-aged patient with nodular goiter due to endemic etiology. After solitary solid nodule formation in the thyroid, autonomous hyperactivity of the nodule finally resulted in clinical hyperthyroidism. Our patient became symptomatic secondary to autonomous adenoma. TH was an incidental finding that was discovered by ultrasound and nuclear scan during evaluation of the patient.

The ultrasound is an imaging modality of choice to assess the structural features of the gland. In our patient, agenesis of one lobe was first established using ultrasound. It also showed the presence of a solitary solid nodule in the remaining right lobe. The nuclear scan is the modality that establishes the functional anatomy of the thyroid. In our case, the functional absence of the left lobe identified by the nuclear scan confirmed its anatomical absence by the ultrasound. The nuclear scan also established the hot nodule in the remaining lobe of our patient. These two imaging modalities are complementary tools to assess the structural and functional features of the thyroid and to establish any anatomical abnormality such as TH in our case. Previous reports have also shown that ultrasound is the first tool for the evaluation of thyroid anatomy [2,8]. Studies have also emphasized the importance of nuclear scan to assess the functional status and to establish the functional abnormality of the gland [8]. Cervical MRI of our patient confirmed TH with the absence of the left lobe. It also revealed the solitary solid nodule in the right lobe.

TH has no specific symptoms and signs leading to the diagnosis of this abnormality. The remaining lobe of the gland generally has a normal function. Usually, patients with TH are biochemically euthyroid and clinically asymptomatic [9]. Most of the cases of TH are diagnosed when the patients present a lesion in the functioning lobe. The remaining lobe of the thyroid gland can be a site of pathological changes similar to a normally developed gland and may present a spectrum of diseases [6]. This anatomical anomaly is generally established during the clinical workup of symptomatic patients with thyroid disorders. In a series of TH cases associated with thyroid diseases, hyperthyroidism constituted only 10% of concomitant disorders of “mono-lobe (unilobate)” gland [2,9]. Autonomous hyperactivity of the gland has clinical significance after symptoms of hyperthyroidism. Therefore, toxic goiter has been reported as the reason for complaints of some patients with TH. Majority of other associated diseases with a normal thyroid function remain asymptomatic during a long period [4]. Thyrotoxic complaints are the primary reason for the evaluation of our patient, wherein biochemical analyses established the diagnosis of hyperactivity.

Hyperthyroidism is surgically treated in the majority of symptomatic cases. Its proper surgical treatment includes the total excision of the hyperactive tissue displayed by the nuclear scan. Hemithyroidectomy, including the hyperactive adenoma, is the surgical procedure of choice in patients with hyperthyroidism due to toxic adenoma. Toxic adenoma cases with a "bilobate" thyroid generally do not require postoperative replacement therapy after hemithyroidectomy that the proper function of the remaining lobe produces and supplies thyroid hormones for maintaining their normal blood levels [10]. Our present patient was also an example of thyrotoxicosis due to toxic adenoma. The absence of the left lobe constituted TH due to which hemithyroidectomy involved the total excision of the thyroidal tissue. She was a "unilobate" TH case, therefore, unilateral exploration was performed and the remaining "only" thyroidal tissue was totally excised. Hemithyroidectomy in TH cases technically becomes a total thyroidectomy with a need for postoperative thyroid replacement therapy. In conclusion, unilateral exploration and total excision of the remaining tissue achieved definitive treatment of thyrotoxicosis.

## Conclusions

TH is a rare abnormality that is usually asymptomatic. The hyperactivity associated with hemiagenesis makes the patient symptomatic. Imaging modalities, ultrasound and nuclear scan establish TH during evaluation of thyrotoxic patients. Total excision of the hyperactive tissue provides definitive treatment of hyperthyroidism. Our patient is a rare case of thyrotoxicosis secondary to toxic adenoma associated with an anatomical abnormality, hemiagenesis.
